# Optimizing sobriety checkpoints to maximize public health benefits and minimize operational costs

**DOI:** 10.1186/s40621-023-00427-8

**Published:** 2023-03-13

**Authors:** Christopher N. Morrison, Ariana N. Gobaud, Christina A. Mehranbod, Brady R. Bushover, Charles C. Branas, Douglas J. Wiebe, Corinne Peek-Asa, Qixuan Chen, Jason Ferris

**Affiliations:** 1 Department of Epidemiology, Mailman School of Public Health, Columbia University, 722 West 168th St, R505, New York, NY 10032 USA; 2 Department of Epidemiology and Preventive Medicine, School of Public Health and Preventive Medicine, Monash University, Melbourne, VIC Australia; 3 Department of Epidemiology, School of Public Health, University of Michigan, Ann Arbor, MI USA; 4 Office of Research Affairs, University of California San Diego, San Diego, CA USA; 5 Department of Biostatistics, Mailman School of Public Health, Columbia University, New York, NY USA; 6 Centre for Health Services Research, The University of Queensland, Woolloongabba, Queensland, Australia

**Keywords:** Drunk driving, Sobriety checkpoints, Optimization, Intervention, Alcohol

## Abstract

**Background:**

Sobriety checkpoints are a highly effective strategy to reduce alcohol-impaired driving, but they are used infrequently in the USA. Recent evidence from observational studies suggests that using optimized sobriety checkpoints—operating for shorter duration with fewer officers—can minimize operational costs without reducing public health benefits. The aim of this research was to conduct a pilot study to test whether police can feasibly implement optimized sobriety checkpoints and whether researchers can examine optimized sobriety checkpoints compared to usual practice within a non-randomized controlled trial study design.

**Methods:**

The study site was the Town of Apex, NC. We worked with Apex Police Department to develop a schedule of sobriety checkpoints during calendar year 2021 that comprised 2 control checkpoints (conducted according to routine practice) and 4 optimized checkpoints staffed by fewer officers. Our primary operations aim was to test whether police can feasibly implement optimized sobriety checkpoints. Our primary research aim was to identify barriers and facilitators for conducting an intervention study of optimized sobriety checkpoints compared to usual practice. A secondary aim was to assess motorist support for sobriety checkpoints and momentary stress while passing through checkpoints.

**Results:**

Apex PD conducted 5 of the 6 checkpoints and reported similar operational capabilities and results during the optimized checkpoints compared to control checkpoints. For example, a mean of 4 drivers were investigated for possibly driving while impaired at the optimized checkpoints, compared to 2 drivers at control checkpoints. The field team conducted intercept surveys among 112 motorists at 4 of the 6 checkpoints in the trial schedule. The survey response rate was 11% from among 1,045 motorists who passed through these checkpoints. Over 90% of respondents supported sobriety checkpoints, and momentary stress during checkpoints was greater for motorists who reported consuming any alcohol in the last 90 days compared to nondrinkers (OR = 6.7, 95%CI: 1.6, 27.1).

**Conclusions:**

Results of this study indicate the sobriety checkpoints can feasibly be optimized by municipal police departments, but it will be very difficult to assess the impacts of optimized checkpoints compared to usual practice using an experimental study design.

## Background

Alcohol-involved motor vehicle crashes have enormous public health costs in the USA. The crash fatality rate due to motor vehicle crashes is greater for the USA than any comparably high-income country (Yellman and Sauber-Schatz 2022), and approximately 19% of all fatal crashes involve some alcohol use (National Center for Statistics and Analysis 2021a). A total of 103,341 people died in alcohol-involved crashes from 2010 to 2019 (Fatality and Injury Reporting System Tool (FIRST) 2021b). There are approximately 8 hospital admissions and 100 emergency department admissions for every one fatal motor vehicle crash in the USA (Centers for Disease Control and Prevention 2014), meaning that many millions of people have been treated for injuries sustained in alcohol-involved crashes in the last decade. Preventive interventions that reduce the relative rate of alcohol-involved crashes by even a small amount compared to the current rate will have important absolute impacts on the burden of injury and death in this country.

Sobriety checkpoints are a preventive intervention with strong empirical support and legal backing as a strategy to reduce alcohol-impaired driving and alcohol-involved crash incidence (Ferris et al. 2013, 2015; Fell et al. 2005). The intervention involves police officers establishing a temporary roadside checkpoint at which they stop drivers to perform a sobriety test. Those who are found to be driving while impaired are prosecuted according to local laws. The intervention was upheld in the US Supreme Court in 1990 (Michigan Department of State Police 1990), when it was determined that law enforcement agencies could conduct sobriety checkpoints, provided they advertised the checkpoint location beforehand and all drivers who passed through the checkpoint were subject to the same sobriety test protocol. Multiple systematic reviews have since found that observational studies provide consistent and compelling evidence that sobriety checkpoints reduce alcohol-impaired driving and alcohol-involved crashes (Shults et al. 2001; Bergen et al. 2014; Erke et al. 2009; Elder et al. 2002; Peek-Asa 1999; Stuster and Blowers 1995). Two experimental studies support these findings, demonstrating that sobriety checkpoints can be included in a suite of community-level interventions to reduce alcohol-involved crashes (Saltz et al. 2021; Voas et al. 1997).

Despite the strong evidence that sobriety checkpoints reduce alcohol-involved crash incidence, the intervention is used infrequently in the USA. Twelve states prohibit sobriety checkpoints by state law or Constitution, and of the 38 states in which sobriety checkpoints are permitted, only 5 conduct any checkpoints more than once per week anywhere in the state (National Highway Traffic Safety Administration 2017; Centers for Disease Control and Prevention 2015). Surveys of law enforcement officers identify several possible reasons for their infrequent use, including perceived ineffectiveness, high operational costs for municipal police departments, and boredom and discomfort for officers (Fell et al. 2003). Research is required to identify ways to optimize sobriety checkpoints by minimizing operating costs while maximizing public health benefits (Community Preventive Services Task Force 2014).

Recent evidence from observational studies and relevant theory suggest that sobriety checkpoints can be optimized. In research conducted in Los Angeles, CA, and Brisbane, Australia, our group detected that individual sobriety checkpoints were associated with a small reduction in alcohol-impaired driving and alcohol-involved crashing for approximately one week over an area roughly equivalent to a small city (~ 60,000 residents) (Morrison et al. 2019). Further, the size and duration of the checkpoints do not affect this association (Morrison et al. 2021). This research highlighted that small checkpoints staffed by few police officers who are in place for short periods have similar impacts compared to large checkpoints staffed by many police officers for long periods. These results are consistent with findings from other researchers in other settings (Nunn and Newby 2011; Voas 2008; Lacey et al. 2006; Lacey and Jones 2000). The primary theoretical explanation for these results is that sobriety checkpoints affect alcohol-impaired driving through general deterrence, wherein the presence of a checkpoint affects perceived risks of detection and punishment for all motorists (Homel 1993). That is, the active ingredient of checkpoints is their promotion and physical presence, not the number of officers, the amount of time they are in place, or the number of alcohol-impaired drivers who are detected. Motorists who become aware of the checkpoint—through in-person experience, social media, gazettes or media advertisements, or via word of mouth—will be less likely to drive while impaired on subsequent occasions (Freeman et al. 2021, 2020). Effects will decay over space as fewer people become aware of the checkpoint and over time as driver behavior regresses to the mean. In combination, this prior work suggests that an overall sobriety checkpoint program could be optimized by conducting a larger number of checkpoints that are staffed by fewer officers for fewer hours each. No experimental studies have examined implementation or impacts of optimized sobriety checkpoints compared to usual practice.

The objective of this research was to conduct a pilot study in one small city. We addressed two primary aims. The primary operations aim was to test whether police can feasibly implement optimized sobriety checkpoints (i.e., smaller checkpoints staffed by fewer officers). The primary research aim was to identify barriers and facilitators for conducting an intervention study of optimized sobriety checkpoints compared to usual practice. A secondary aim was to assess motorist support for sobriety checkpoints and momentary stress while passing through checkpoints. Given the small scale and limited scope, outcomes of interest were process measures (e.g., following protocols) rather than public health impact measures (e.g., reductions in alcohol-impaired driver crashes).

## Methods

### Study setting

**Fig. 1:**
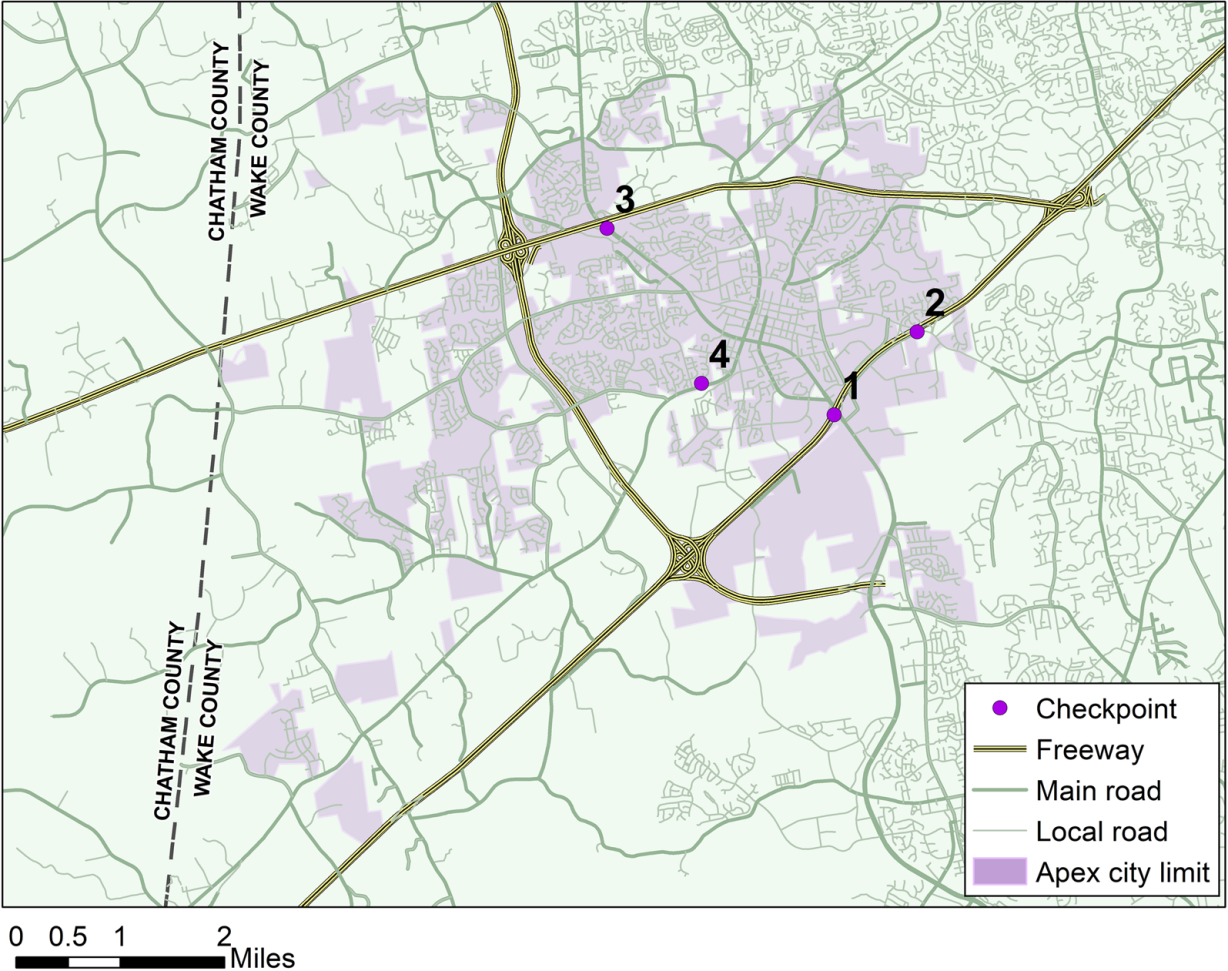
Township of Apex, North Carolina

The setting for this study was the Town of Apex, North Carolina, which is in the greater metropolitan area of Raleigh-Durham, Wake County, and is located 15 miles southeast of downtown Raleigh. The town has a population of 71,988, covers a land area of 21.5 miles^2^, and is transected by three major freeways that surround a small historic business and residential district (Fig. 1). The median household income for Apex in 2019 was $111,435, which was 1.6 times greater than the median household income for the USA overall.

Apex was selected as the pilot study site in consultation with the National Police Foundation (Arlington, VA), because Apex Police Department (PD) operates a consistent schedule of sobriety checkpoints and has been an active participant in the development of road safety interventions. The department allocates 96 sworn full-time officers and is a member of the Wake County Traffic Safety Taskforce, a collaboration between the North Carolina State Highway Patrol, Wake County Sheriff’s Office and 13 municipal police departments in that area. Apex PD is also an active participant in the North Carolina Governor’s Highway Safety Program (NCGHSP) “Booze It & Lose It” campaign. NCGHSP supports police departments to conduct holiday blitzes to enforce drunk driving laws, including providing a mobile breath testing bus that can accommodate a magistrate for rapid processing of DWI (driving while impaired) charges (North Carolina Department of Transportation 2022). In 2021, the campaign supported 850 sobriety checkpoints in NC, including 15 in Wake County.

### Study design

The intervention condition was optimized sobriety checkpoints configured to maximize public health benefits while minimizing operational costs. The control condition was sobriety checkpoints conducted according to routine practice. This pilot of a non-randomized controlled trial was approved by the Columbia University Institutional Review Board.

**Fig. 2:**
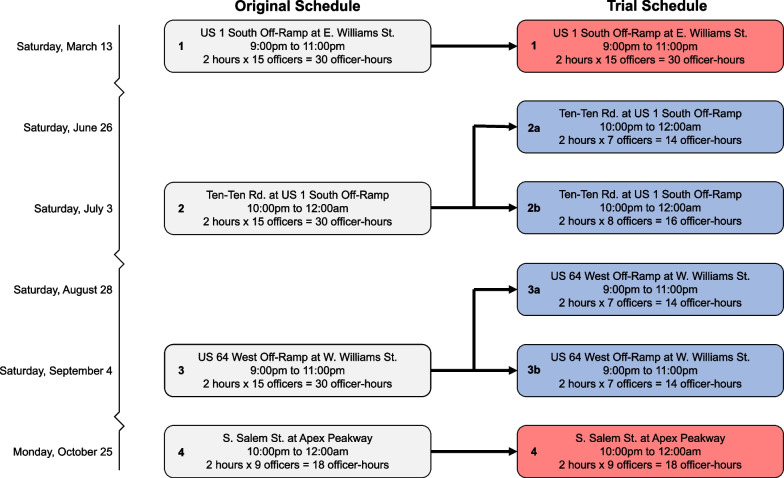
A schedule of intervention checkpoints (blue) and control checkpoints (red) was developed based on the schedule of checkpoints originally prepared by Apex Police Department

In December 2020, in collaboration with the authors and the National Police Foundation (Arlington, VA) and following standard police agency protocols, Apex PD prepared a list of 4 sobriety checkpoints to be conducted during the 2021 calendar year. Checkpoint schedules included the location (street address and nearest cross-street), start time, end time, and number of officers assigned. In consultation with Apex PD, we purposively selected 2 of these routine checkpoints for optimization. These 2 checkpoints were replaced with 4 shorter and smaller checkpoints, such that the costs (operationalized as total officer-hours) of conducting the 4 optimized checkpoints were the same as the costs for the 2 originally scheduled checkpoints. For each of the checkpoints that were optimized, one checkpoint was scheduled at the same time and location as the original checkpoint, and the additional optimized checkpoint was conducted 1 week before that date at the same location. The 2 control checkpoints were conducted according to the original schedule following routine procedures. Figure 2 shows the original checkpoint schedule and the trial checkpoint schedule. For example, checkpoint #2 was originally scheduled at the US 1 South off-ramp and Ten-Ten Road on Saturday July 3 from 10:00 pm to 12:00am with 15 officers. We split the 30 officer-hours into two checkpoints conducted at the same location on Saturday August 28 and Saturday July 3, such that total personnel cost was the same but an additional checkpoint day was included. We met regularly with Apex PD senior command to exchange information about all stages of the study.

### Operations outcomes

To address the primary operations aim—to test whether police can feasibly implement optimized sobriety checkpoints—we used process data provided by Apex PD. Specifically, we accessed information describing the number of officers who staffed the checkpoint (i.e., capturing fidelity to the study design), payroll costs per checkpoints, counts of DWI investigations, and counts of DWI arrests. We also reviewed the after-action reports prepared by Apex PD senior command describing the activities at each checkpoint.

### Research outcomes

The research outcomes—to assess researchers’ ability to examine optimized sobriety checkpoints compared to usual practice within a controlled trial study design—were based primarily on an intercept survey of motorists who passed through the sobriety checkpoints. Police officers interacted with all drivers who passed through the checkpoints according to routine practice (including conducting license checks and initial assessments of probable impairment). Those not stopped for possible violations were invited to continue through the checkpoint and stop at a research station positioned along the roadway at the end of the checkpoint. At the research station, members of a staff field team engaged with drivers to recruit participants. Eligible participants were aged ≥ 21 years, could read English, and had a smartphone. Drivers remained in their vehicles and the research assistants obtained written informed consent from eligible drivers who accepted the invitation to participate in the data collection. Research assistants then provided access to an online survey through display of a QR code, and participants completed the survey on their own mobile devices. The survey took approximately 7 min to complete. Participants received a $20 online shopping voucher.

### Survey data

The intercept survey included measures of *momentary perceived stress* and *support for sobriety checkpoints*. Momentary perceived stress was assessed by self-report with the question, “How stressful is driving through a sobriety checkpoint for you?” Responses were recorded as an integer that ranged from 0 (not stressful at all) to 100 (extremely stressful). We assessed support for sobriety checkpoints using items adapted from the 2009 Traffic Safety Culture Index, conducted by the AAA Foundation for Traffic Safety (AAA Foundation for Traffic Safety 2009). Participants were asked “Do you support sobriety checkpoints in which police officers test all drivers who come through a certain place in your community?”. We also asked whether they supported sobriety checkpoints being conducted in their community at different frequencies (once every few months; several times every month).

We collected self-reported information regarding alcohol-impaired driving using items adapted from the Form 90-DWI sub-scale (Miller and Boca 1994; Usdan et al. 2002; Hettema et al. 2008). Participants provided two self-reported measures of impaired driving by responding to the questions “Have you driven after drinking any alcohol in the past 90 days” (driving after drinking) and “Have you driven after drinking too much alcohol in the past 90 days” (drunk driving). We also measured the frequency of alcohol consumption in the previous 90 days (never, monthly or less [“monthly”], 2–4 times per month [“weekly”], ≥ 2 times per week [“daily”]). To account for the possibility that momentary perceived stress was affected by prior events, we measured prior stress as, “Thinking about your day before you drove through this sobriety checkpoint: Did anything stressful occur today? A stressful event is any event, even a minor one, which negatively affected you.” Participants also reported demographic and economic characteristics (e.g., age [years], race/ethnicity [Non-Hispanic White, Non-Hispanic Black, Hispanic, Other], annual income [categorical]).

### Statistical analysis

To assess whether police can feasibly implement optimized sobriety checkpoints, we observed whether Apex PD were able to conduct the planned checkpoints and compared descriptive statistics for police operations data for intervention compared to control checkpoints. To address the primary research aim—identifying barriers and facilitators for conducting an intervention study of optimized sobriety checkpoints compared to usual practice—we measured the response rate for the intercept survey as the number of complete survey responses divided by the number of people invited to participate, per American Association of Public Opinion Researchers standard definitions (Standard Definitions: Final Dispositions of Case Codes and Outcome Rates for Surveys 2016).

To address the secondary aim—to assess motorist support for sobriety checkpoints and momentary stress while passing through checkpoints—we examined predictors of momentary stress and support for sobriety checkpoints in two logistic regression models. Exposures of interest were motorists’ demographic, economic, and behavioral characteristics. The model for momentary stress controlled for prior stress, and the model for supporting checkpoints controlled for driving after drinking in the prior 90 days.

## Results

Apex Police Department successfully completed 5 of the 6 checkpoints in the trial schedule (Table 1). Checkpoint #4 was not conducted due to inclement weather, meaning that 4 intervention checkpoints (#2A, #2B, #3A, and #3B) and 1 control checkpoint (#1) were conducted in accordance with the trial schedule. The intervention checkpoints were operational for 2 h each and had 8 officers in attendance; the control checkpoint was operational for two hours and had 14 officers in attendance. Total staffing costs for control Checkpoint #1 were $701.06; mean staffing costs for the intervention checkpoints were $531.08. Approximately 8 volunteers from the community group, Citizens Assisting Police in Apex (CAPA), provided practical support at each checkpoint by helping to direct traffic, offering Spanish language interpretation, and moving the vehicles of motorists who were detained.

**Table 1:** Police and research operations outcomes

Checkpoint	1	2a	2b	3a	3b	4
Checkpoint type	Control	Intervention	Intervention	Intervention	Intervention	Control
Total vehicles	n/a	235	294	290	232	n/a
Total vehicles stopped	n/a	235	294	284	232	n/a
Police operations						
Conducted at planned location	Yes	Yes	Yes	Yes	Yes	No
Conducted at planned hours	Yes	Yes	Yes	Yes	Yes	No
Officers present	14	8	8	8	8	0
Total payroll costs	$701.06	$547.43	$524.29	$557.08	$495.51	n/a
Research operations						
Conducted field data collection	No	Yes	Yes	Yes	Yes	No
Research assistants present	0	4	4	5	5	0
DWI outcomes						
DWI investigation	2	1	6	4	1	–
DWI arrests	0	1	0	0	0	–
DWI convictions	0	0	0	0	0	–
Open container	1	1	0	0	0	–
DWI over 21	0	1	0	0	0	–
Other outcomes						
No operator’s license	4	15	11	7	7	–
DWLR violations	8	3	4	1	4	–
Registration violations	3	1	2	3	1	–
Other violations	19	4	3	3	2	–
Warnings	3	1	1	5	0	–
Drug violations	0	0	1	1	0	–
Wanted persons	0	0	0	0	0	–
Total	37	24	22	20	14	–

### Primary operations aim

The primary operations aim was to test whether police can feasibly implement optimized sobriety checkpoints. Operations were mostly similar for the intervention checkpoints compared to the control checkpoint. Officers conducted between 1 and 6 DWI investigations during the intervention checkpoints and conducted 2 DWI investigations during the control checkpoint. They made 1 DWI arrest during checkpoint #2A based on clues detected during Standardized Field Sobriety Testing (SFST). The driver submitted to a breath test, which resulted in a blood alcohol content (BAC) below the threshold established by state law and was arrested in accordance with applicable standards. Other outcomes (e.g., registration violations) occurred more frequently in the control checkpoint (total = 37) compared to the intervention checkpoints (range = 14 to 24). After-action reports from Apex PD senior command described no difficulties related to traffic flow, staffing, or feasibility in any of the 5 checkpoints.

### Primary research aim

The primary research aim was to identify barriers and facilitators for conducting an intervention study of optimized sobriety checkpoints compared to usual practice. The field team of research assistants completed data collection at 4 of the 6 checkpoints. Apex PD conducted checkpoint #1 as planned but the field team could not attend the site due to travel restrictions related to COVID-19. A team of 4 to 5 research assistants attended each of the 4 intervention checkpoints and conducted field data collection in accordance with the study protocol. A total of 1,051 motorists passed through the 4 checkpoints, of whom 1,045 underwent a field sobriety test (6 vehicles were allowed to pass through checkpoint #3A to provide passage for an emergency vehicle), and 1,040 were invited to participate in the survey. A total of 147 attended the survey station, 125 met the eligibility criteria and agreed to participate in the survey, and 112 completed the survey. The overall response rate was 11%, assuming all non-respondents were eligible (Table 2).

**Table 2:** Response rate

Checkpoint	1	2a	2b	3a	3b	4	Total
Passed through Checkpoint	–	235	294	290	232	–	1051
Stopped for Sobriety Test	–	235	294	284	232	–	1045
Stopped for Suspected DUI	–	1	1	0	0	–	2
Invited to Participate in Survey (I)	–	234	293	283	230	–	1040
Attended Survey Station	–	34	39	39	35	–	147
Began Survey	–	28	32	33	32	–	125
Completed Survey (C)	–	26	28	30	28	–	112
*Response Rate* [(C ÷ I) × 100]	*–*	11%	10%	11%	12%	–	11%

### Secondary aim

The secondary aim was to assess motorist support for sobriety checkpoints and momentary stress while passing through checkpoints. Of the 112 motorists who completed the survey, 101 (90%) supported having any sobriety checkpoints in their community and 76 (68%) supported having checkpoints in their community several times every month (Table 3). On a scale from 0 to 100, perceived momentary stress attributable to checkpoints had a bimodal distribution with a mean of 22.4 (median = 1; IQR = 0, 50). We were not able to examine whether checkpoint configuration affected motorists’ perceptions because we did not collect any survey data during the control checkpoints (#1 and #4). However, the available data enabled us to relate motorist characteristics to perceived momentary stress and support for checkpoints. In logistic regression models, motorist demographic, economic, and behavioral characteristics were not related to support for having checkpoints in the community several times every month (Table 4). Motorists who consumed alcohol weekly had 14 times increased odds of reporting that sobriety checkpoints were stressful compared to motorists who did not consume alcohol (aOR = 14.3, 95%CI: 3.0, 69.6, *p* < 0.001). Notably, of the 28 people who reported no alcohol consumption, almost all (*n* = 25) reported that checkpoints were no or low stress. Likelihood ratio tests provided no evidence that model fit was better after adding random effects for checkpoints (*p* > 0.5).

**Table 3:** Participant characteristics (*n* = 112)

Variable	*n*	%
Dependent measures		
Momentary perceived stress (above the sample mean)	37	33.0
Support for sobriety checkpoints		
Any	101	90.2
Once every few months	101	90.2
Several times every month	76	67.9
Independent measures		
Prior stress	23	20.5
Drove after drinking in last 90 days	21	18.8
Drunk driving in last 90 days	0	0.0
Alcohol consumption		
None	29	25.9
Monthly	29	25.9
Weekly	24	21.4
Daily	30	26.8
Male	60	53.6
Age (quartiles)		
21–28 years	29	25.9
29–39 years	29	25.9
40–49 years	24	21.4
50–75 years	30	26.8
Race/ethnicity		
Non-Hispanic White	65	58.0
Non-Hispanic Black	18	16.1
Hispanic	10	8.9
Other	19	17.0
Income		
< $40 k	17	15.2
$40–80 k	37	33.0
$80–120 k	19	17.0
> $120 k	33	29.5
Chose not to answer	6	5.4

**Table 4:** Logistic regression models (*n* = 112)

	Support for Sobriety Checkpoints (Several Times Every Month)				Momentary Perceived Stress			
	OR	(95%	CI)	*p*-value	OR	(95%	CI)	*p*-value
Prior stress					1.02	0.31	3.28	0.980
Drove after drinking in last 90 days	0.55	0.16	1.84	0.332				
Alcohol consumption								
None [ref]								
Monthly	0.40	0.11	1.40	0.150	3.88	0.85	17.77	0.081
Weekly	0.42	0.11	1.67	0.218	14.35	2.96	69.57	0.001
Daily	0.31	0.06	1.67	0.174	5.67	0.89	35.91	0.066
Male	1.23	0.51	2.97	0.647	0.62	0.24	1.64	0.338
Age								
21–28 years [ref]								
29–39 years	1.71	0.45	6.46	0.430	0.51	0.12	2.19	0.363
40–49 years	1.42	0.40	5.01	0.583	0.78	0.20	3.11	0.724
50–75 years	1.66	0.44	6.33	0.458	0.50	0.12	2.18	0.359
Race/ethnicity								
Non-Hispanic White [ref]								
Non-Hispanic Black	0.70	0.20	2.49	0.585	0.71	0.18	2.81	0.625
Hispanic	0.85	0.17	4.35	0.850	0.53	0.07	3.86	0.529
Other	0.45	0.12	1.78	0.257	1.55	0.34	7.09	0.569
Income								
< $40 k [ref]								
$40–80 k	0.81	0.21	3.16	0.759	1.53	0.36	6.56	0.567
$80–120 k	1.27	0.26	6.21	0.770	0.19	0.03	1.39	0.103
> $120 k	0.76	0.18	3.17	0.704	1.05	0.22	4.90	0.954
Chose not to answer	2.20	0.16	30.09	0.554	1.35	0.13	14.02	0.800

## Discussion

Alcohol-involved motor vehicle crashes have considerable public health costs in the USA, and sobriety checkpoints are a highly effective but underutilized intervention that could substantially reduce this burden (Voas and Fell 2013). Guided by prior findings that operational costs to municipal police departments are an impediment to checkpoint implementation (Fell et al. 2003), and that smaller and shorter checkpoints are similarly effective compared to larger and longer checkpoints (Morrison et al. 2019, 2021, 2022), we tested whether it was feasible to implement and study optimized checkpoints to maximize public health benefits while minimizing operational costs. Results were mixed. We found that checkpoints can be optimized and that municipal police departments can achieve comparable operational outcomes using checkpoints with fewer officers on-site. But we also identified important obstacles to empirical study of the intervention, including that practical considerations can prevent planned research and law enforcement activities, and that the intercept surveys of motorists can yield very low response rates.

Our primary operations aim was to assess whether police could feasibly implement optimized sobriety checkpoints. We found that policing operations in four optimized checkpoints were similar to operations in one control checkpoint, providing preliminary evidence to support future efforts to optimize sobriety checkpoints within US police departments. The comparable number of DWI outcomes (e.g., investigations) and other outcomes (e.g., no operator’s license), and the unimpeded traffic flow for intervention compared to control checkpoints suggests that conducting checkpoints with fewer staff is manageable. Our ongoing discussions with senior officers at Apex PD revealed no other obstacles to ongoing use of optimized checkpoints. It is therefore possible that other police departments around the USA will be able to achieve similar operational cost savings. However, important caveats apply. The financial burden of conducting two small checkpoints were greater than conducting one large checkpoint due to fixed costs (e.g., field briefings, planning, advertising), meaning that the operational cost of the trial schedule exceeded the operational cost of the original schedule. Additionally, the presence of Citizens Assisting Police in Apex—who provided essential support for Apex PD officers—may limit generalizability to other contexts. Other police departments that implement an optimized schedule but do not have this form of logistical support may have difficulty achieving similar operations outcomes compared to Apex PD.

Our primary research aim was to assess whether researchers could examine optimized sobriety checkpoints compared to usual practice within a controlled trial study design. We found that future research conducted at a larger scale will be difficult to implement. Apex PD engaged readily with the research team through the study and adhered diligently to the agreed trial schedule of sobriety checkpoints. They accommodated field data collection teams on-site at the checkpoints, provided access to after-action reports, and engaged in bidirectional flow of information regarding checkpoint operations and study findings. However, we identified that researchers seeking to compare optimized sobriety checkpoints to usual practice must contend with other important obstacles. Non-adherence to the trial schedule for Apex PD (checkpoint #1) and the field research team (checkpoints #1 and #4) due to COVID-19 and inclement weather are examples of everyday complications could affect implementation research in any setting. The poor response rate for the intercept survey suggests that future research should not rely on this method to produce generalizable information about the population of motorists who pass through a checkpoint. Intercept surveys are a common approach for studying motorist knowledge, attitudes, and behaviors (Jamt et al. 2019; Pollini et al. 2017); however, it appears the method is poorly suited for implementation in the context of a sobriety checkpoint. Quantitative analyses of checkpoint effects must therefore be conducted on aggregate (e.g., in ecological analyses comparing crash incidence for municipalities that receive optimized checkpoints to municipalities that receive control checkpoints); however, the small effect sizes for individual checkpoints mean that it may not be practicable to detect intervention effects at a population level (Morrison et al. 2019, 2021).

Notwithstanding the results of the primary study aims, our secondary analysis of the motorist intercept survey data contains some useful information to help guide the design of checkpoint programs. We find no reason to expect that motorist perceptions would be influenced by the length of the checkpoint or the number of officers present. The strong support for sobriety checkpoints among respondents and the null associations between driver characteristics and sobriety checkpoint support partially corroborate prior findings that checkpoints are well-accepted interventions within communities (North Carolina Department of Transportation 2022). The clear limitation is that non-respondents may be less supportive than respondents. Perhaps a more compelling finding is that drinkers report that checkpoints are more stressful than nondrinkers. In accordance with general deterrence theory (Lacey and Jones 2000), motorists who drink alcohol and who learn that a checkpoint was conducted may change their subsequent behavior, whereas nondrinkers could be relatively unaffected. Failing to complete the field data collection in the control checkpoints (#1 and #4) meant we could not evaluate treatment effects.

This research has important limitations that may affect generalizability to other contexts. Conducting the study in a higher-income township with a municipal police department that was strongly supportive of DWI interventions and a senior command who were amenable to research may lead to an unrealistic assessment of the feasibility of implementing optimized sobriety checkpoints. For example, few municipal police departments have community volunteer organizations such as Citizens Assisting Police in Apex to help implement these interventions, which may lead to unrealistically optimistic assessment of the feasibility of conducting optimized checkpoints. As noted above, the non-generalizable sample of motorists means the results of from survey data should be interpreted with caution. We were not able to assess characteristics for non-respondents due to concerns about misclassification (e.g., observed race and ethnicity), but it is possible that non-respondents differed systematically compared to respondents. A further essential consideration is concern related to systematic bias in relation to the motorists whom police select for field sobriety tests and potential impacts of sobriety checkpoints on police–community relations (cite). The Supreme Court judgment from Michigan Department of State Police et al. v. Sitz et al. requires that police do not profile motorists during sobriety checkpoints. However, we were not able to determine whether motorist support differed for intervention compared to control checkpoints and this small pilot study was not designed to assess broader community-level impacts.

## Conclusions

Theory and empirical studies provide compelling evidence that sobriety checkpoints reduce alcohol-involved crashes. Given the considerable public health toll that alcohol-involved crashes have in the USA, the imperative for research is to move beyond intervention studies that test whether these associations are present, to implementation studies that examine how to apply the intervention most effectively in real-world settings. This research provides preliminary confirmation that one municipal police department was able to feasibly conduct additional sobriety checkpoints staffed by fewer officers without impeding law enforcement activities. However, it may not be possible to test intervention effects within a rigorous scientific framework.

## Data Availability

The datasets used and/or analyzed during the current study are available from the corresponding author on reasonable request.

## Abbreviations

NCGHSP: North Carolina Governor’s Highway Safety Program
DWI: Driving while impaired
PD: Police department
CAPA: Citizens Assisting Police in Apex
SFST: Standardized Field Sobriety Testing
BAC: Blood alcohol content
